# Evidence that the *Plasmodium falciparum* Protein Sortilin Potentially Acts as an Escorter for the Trafficking of the Rhoptry-Associated Membrane Antigen to the Rhoptries

**DOI:** 10.1128/mSphere.00551-17

**Published:** 2018-01-03

**Authors:** Stéphanie Hallée, Justin A. Boddey, Alan F. Cowman, Dave Richard

**Affiliations:** a Centre de Recherche en Infectiologie du CHU de Québec-Université Laval, Quebec City, Quebec, Canada; b The Walter and Eliza Hall Institute of Medical Research, Parkville, Victoria, Australia; c Department of Medical Biology, University of Melbourne, Parkville, Victoria, Australia; Johns Hopkins Bloomberg School of Public Health

**Keywords:** malaria, sortilin, protein trafficking

## Abstract

The rhoptry organelle is critical for the invasion of an erythrocyte by the malaria parasite *Plasmodium falciparum*. Despite their critical roles, the mechanisms behind their biogenesis are still poorly defined. Our earlier work had suggested that the interaction between the glycosylphosphatidylinositol (GPI)-anchored rhoptry-associated membrane antigen (RAMA) and the soluble rhoptry-associated protein 1 was involved in the transport of the latter from the Golgi apparatus to the rhoptry. However, how this protein complex could interact with the intracellular trafficking machinery was unknown at this stage. Here we show that the *P. falciparum* homologue of the transmembrane protein sortilin-VPS10 interacts with regions of RAMA that are sufficient to target a fluorescent reporter to the rhoptries. These results suggest that *P. falciparum* sortilin (PfSortilin) could potentially act as the escorter for the transport of rhoptry-destined cargo.

**IMPORTANCE** The malaria parasite is a massive burden in several parts of the world. Worryingly, the parasite has become resistant to several of the drugs commonly used to treat the disease, and at this time, there is no commercial vaccine. It is therefore critical to identify new targets for the development of antimalarials. To survive in the human body, the malaria parasite needs to invade red blood cells. For this, it uses a variety of effectors stored in organelles forming a structure called the apical complex. The mechanisms behind how the parasite generates the apical complex are poorly understood. In this study, we present evidence that a transmembrane protein called sortilin potentially acts as an escorter to transport proteins from the Golgi apparatus to the rhoptries, a component of the apical complex. Our study provides new insight into the biogenesis of a critical structure of the malaria parasite.

## Abstract (precis)

The malaria parasite is a massive burden in several parts of the world. Worryingly, the parasite has become resistant to several of the drugs commonly used to treat the disease, and at this time, there is no commercial vaccine. It is therefore critical to identify new targets for the development of antimalarials. To survive in the human body, the malaria parasite needs to invade red blood cells. For this, it uses a variety of effectors stored in organelles forming a structure called the apical complex. The mechanisms behind how the parasite generates the apical complex are poorly understood. In this study, we present evidence that a transmembrane protein called sortilin potentially acts as an escorter to transport proteins from the Golgi apparatus to the rhoptries, a component of the apical complex. Our study provides new insight into the biogenesis of a critical structure of the malaria parasite.

## INTRODUCTION

Despite great progress in reducing the mortality and morbidity of malaria over the past years, the disease still represents an enormous burden in several tropical and subtropical regions of the globe. In 2015, more than 430,000 deaths were caused by *Plasmodium* parasites, and most of these were due to *Plasmodium falciparum*, which is responsible for the most severe form of malaria (1). Resistance to most currently available antimalarials, including the first-line drug artemisinin (2), and the absence of a sterilizing vaccine demonstrate the need to develop novel intervention strategies.

Like all apicomplexans, *Plasmodium* spp. are obligate intracellular parasites, and their life cycle is initiated by invasion of their target host cell. Invasion of the erythrocyte by the malaria merozoite is a multistep process driven by the highly coordinated sequential release of organelles forming the apical complex: the rhoptries, micronemes, and dense granules (3). These organelles are formed *de novo* during a peculiar cell division process termed schizogony (4). Tremendous progress in the unraveling of intracellular protein trafficking in the model apicomplexan *Toxoplasma gondii* has led to the concept of an evolutionary repurposing of the endosomal systems for the biogenesis of rhoptries and micronemes (5). In comparison, the mechanisms driving the biogenesis of the apical complex in *P. falciparum* are poorly defined, although evidence pointing to a direct route from the Golgi apparatus suggests that intermediate endosome-like compartments as found in *T. gondii* might not be required (6–8). Recent studies showing partial colocalization of the *P. falciparum* homologues of the small G-protein Rab11A and of adaptor protein 1 with markers of the rhoptry has led to the suggestion that these proteins might be involved in the process of vesicular fusion at the rhoptry membrane (9, 10).

Our previous results had suggested that the glycosylphosphatidylinositol (GPI)-anchored *P. falciparum* rhoptry protein rhoptry-associated membrane antigen (RAMA) (PF3D7_0707300) acted as an escorter for several other rhoptry proteins that exist in a low-molecular-weight rhoptry complex termed the rhoptry-associated protein (RAP) complex. This led us to propose a model whereby differential sorting to the apical complex organelles involves the aggregation of multiprotein complexes in distinct subdomains of the Golgi membrane (11). Central to this hypothesis was the requirement of putative organelle-specific transmembrane escort proteins which would package rhoptry-, microneme-, or dense-granule-destined cargo into distinct transport vesicles. However, *P. falciparum*, like other apicomplexan parasites, does not possess a mannose-6-phosphate receptor, which recognizes proteins that have been tagged with mannose-6-phosphate groups in the Golgi apparatus and packages them into transport vesicles for delivery to endosomal/lysosomal compartments (12, 13). Sortilin proteins, which are known by the alternate name VPS10p in yeast cells, have a conserved structure consisting of an N-terminal propeptide, a VPS10 domain for binding to cargo proteins, a transmembrane domain, and finally a cytoplasmic tail interacting with the intracellular trafficking machinery (14). In yeast, VPS10p is involved in trafficking of hydrolases to the vacuole (15), while in mammalian cells, it acts as an escorter to transport proteins to the plasma membrane, endocytic pathway, and lysosomes but also serves as a cell surface receptor (16). Recent work has identified an *P. falciparum* homologue of the sortilin protein (PF3D7_1451800) and suggested that it was playing a role in cargo shuttling between the endosome and the Golgi apparatus (17). Because of the conserved role of sortilin homologues as protein escorters, we were therefore interested in exploring the possibility that *P. falciparum* sortilin (PfSortilin) was involved in the targeting of proteins from the Golgi apparatus to the rhoptries.

Here, we present the characterization of the *P. falciparum* homologue of sortilin. We show that it localizes to the *cis* region of the Golgi apparatus and interacts with regions of RAMA that are sufficient for correct trafficking to the rhoptries. We therefore propose that PfSortilin potentially acts as an escorter to transport the RAMA-RAP protein complex from the Golgi apparatus to the rhoptries.

## RESULTS AND DISCUSSION

### *P. falciparum* sortilin localizes to the *cis* region of the Golgi apparatus throughout the erythrocytic cycle.

**FIG 1:**
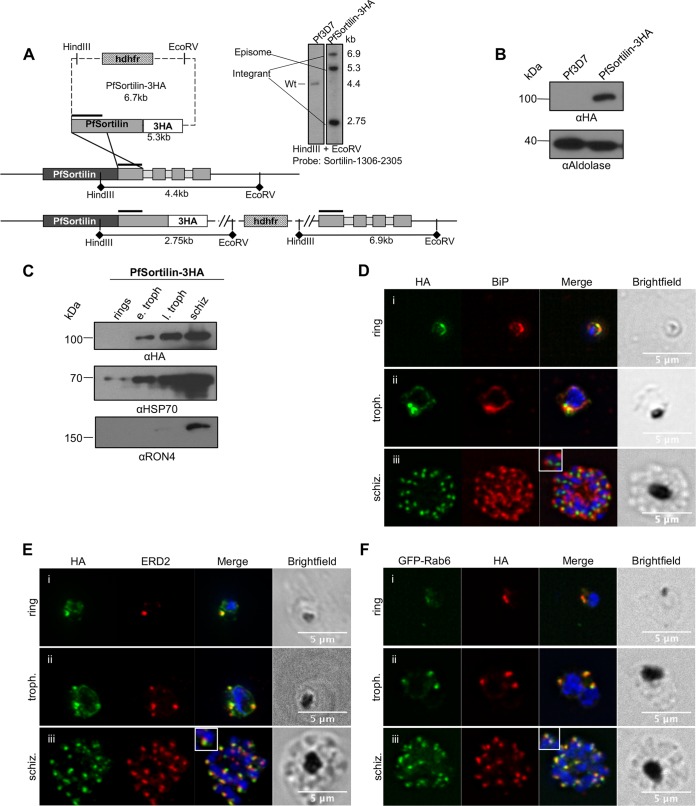
Generation of the 3HA-tagged PfSortilin line. (A) Schematic of the knock-in strategy and Southern blot showing proper integration of the plasmid and the disappearance of the WT allele. Pf3D7, *P. falciparum* 3D7. (B) Western blot showing a specific band at the expected size of around 100 kDa for PfSortilin-3HA. αHA, anti-HA antibody. (C) Time course of expression of PfSortilin-3HA. HSP70 and RON4 are used as staging controls. e. troph, early trophozoites; l. troph, late trophozoites; schiz, schizonts. (D) IFA showing that PfSortilin-3HA is expressed throughout the erythrocytic cycle and partially overlaps with the ER marker BiP in rings and trophozoites. (E) IFA showing extensive colocalization between PfSortilin-3HA and the *cis*-Golgi marker ERD2 at all stages. (F) IFA showing extensive colocalization between PfSortilin-3HA and the *trans*-Golgi marker GFP-Rab6 at all stages.

To determine the subcellular localization of PfSortilin, we tagged the endogenous gene at the 3′ end with a triple-hemagglutinin (3HA) tag by single-crossover recombination (Fig. 1A). Western blots on mixed-stages parasite extracts of a PfSortilin-3HA clonal line revealed a single band at the expected size of around 100 kDa (Fig. 1B). To determine the expression profile of PfSortilin throughout the erythrocytic cycle, we performed Western blotting on tightly synchronized parasites taken at different stages of the cycle. This revealed that the protein was detected from early trophozoite stage through schizogony as previously described (Fig. 1C) (17). Antibodies against the constitutive protein HSP70 and the schizont protein RON4 were used as staging controls (Fig. 1C).

**FIG 2:**
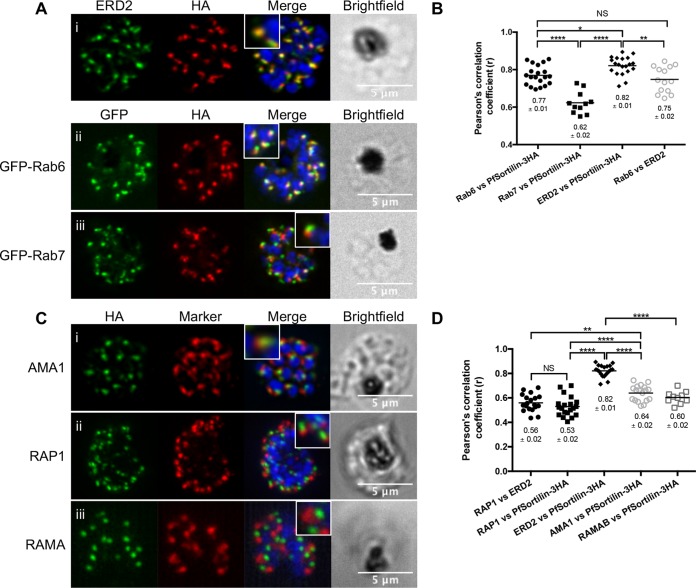
*Plasmodium falciparum* sortilin localizes to the *cis* region of the Golgi apparatus. (A) IFA showing extensive colocalization between PfSortilin-3HA and the *cis*-Golgi marker ERD2. Some overlap is also seen with the *trans*-Golgi marker GFP-Rab6, while little overlap is obtained with the late endosome marker GFP-Rab7. (B) Pearson’s correlation analysis demonstrates that PfSortilin-3HA overlaps significantly more with ERD2 than with either Rab6 or Rab7. Each symbol represents the value for an individual cell. The numbers of cells analyzed (*n*) are as follows: Rab6 versus PfSortilin-3HA and ERD2 versus PfSortilin-3HA, *n* = 20; Rab7 versus PfSortilin-3HA, *n* = 11; Rab6 versus ERD2, *n* = 14. (C) IFA showing that PfSortilin-3HA does not colocalize with either the micronemal marker AMA1 or the rhoptry markers RAP1 and RAMA. (D) Pearson’s correlation analysis to confirm that PfSortilin-3HA overlaps significantly more with ERD2 than either AMA1, RAP1, or RAMA. PfSortilin-3HA versus RAP1 and PfSortilin-3HA versus ERD2, *n* = 20; RAP1 versus ERD2, *n* = 19; PfSortilin-3HA versus AMA1, *n* = 18. PfSortilin-3HA versus RAMA, *n* = 10. Values that are significantly different are indicated by bars and asterisks as follows: *, *P* < 0.05; **, *P* < 0.01; ****, *P* < 0.0001. Values that are not significantly different (NS) are also indicated. *P* values were calculated using one-way ANOVA followed by a Tukey’s multiple-comparison test. Values shown below the symbols in panels B and D are the means ± standard errors.

We next looked at the subcellular distribution of PfSortilin by immunofluorescence assays (IFA). In ring stages, we could clearly see a distinct focus of fluorescence, along with a perinuclear signal (Fig. 1Di). This pattern was also seen in trophozoites, and some partial colocalization with an anti-binding immunoglobulin protein (anti-BiP) antibody demonstrated that a portion of the protein was found in the endoplasmic reticulum (ER) (Fig. 1Di and ii). In schizont-stage parasites, the fluorescence focus had multiplied and no longer colocalized with BiP, reminiscent of the behavior of other proteins present in the Golgi apparatus during schizogony (Fig. 1Diii) (18–20). Extensive colocalization with markers of the *cis*-Golgi (*e*ndoplasmic reticulum *r*etention-*d*efective ERD2) and the *trans*-Golgi (Rab6) confirmed that PfSortilin was indeed localized to the Golgi apparatus as previously shown (17) (Fig. 1E and F and Fig. 2Ai and ii). Quantification of the level of colocalization revealed that PfSortilin overlapped more with ERD2 than Rab6 (*R* coefficient of 0.82 ± 0.01 for PfSortilin-ERD2 compared to 0.77 ± 0.01 for PfSortilin versus Rab6), which suggests that PfSortilin localizes to the *cis*-Golgi. This is further supported by the *R* coefficient obtained for the *cis*- and *trans*-Golgi markers ERD2 and Rab6, which is similar to PfSortilin versus Rab6 (0.75 ± 0.02 versus 0.77 ± 0.01).

Since sortilin is known to cycle between the *trans*-Golgi and endosomes in yeast and mammalian cells (21) and the related apicomplexan *Toxoplasma gondii* sortilin (TgSortilin) partially colocalizes with the endosome-like compartments (22), we consequently determined whether the same was true in *P. falciparum* by colocalization with Rab7, a marker of the late endosome (17, 23). Our results showed that despite being very close, the two signals never overlapped in schizont stages, and this was corroborated by an *R* coefficient significantly lower than for PfSortilin and the Golgi markers (Fig. 2Aiii and C). This suggests that the major proportion of PfSortilin is found at the *cis*-Golgi apparatus in schizont-stage parasites. We next attempted to perform immunoelectron microscopy (IEM) to determine whether a portion of PfSortilin could be found in endosome-like structures in addition to the Golgi apparatus, as has been observed with TgSortilin (22); however, we were not successful in obtaining specific labeling. It is worth mentioning that, to our knowledge, no protein from either the Golgi apparatus or putative endosome-like organelles have ever been successfully detected by IEM in *P. falciparum*.

We next investigated whether PfSortilin colocalized with micronemal and rhoptry markers as is the case for TgSortilin (22). Minimal overlap was seen with either the micronemal protein AMA1 or the rhoptry bulb proteins RAP1 and RAMA (Fig. 2C). As a control for proteins residing in different organelles, we calculated the colocalization coefficient for ERD2 and RAP1 and obtained a similar value as for PfSortilin versus RAP1 (0.56 **±** 0.02 versus 0.53 to 0.02, respectively; Fig. 2D). In conclusion, our results show that PfSortilin is localized to the ER and Golgi apparatus in rings and trophozoites, redistributing to the *cis* region of the Golgi apparatus in schizonts.

### RAMA interacts with PfSortilin for localization to the rhoptries.

**FIG 3:**
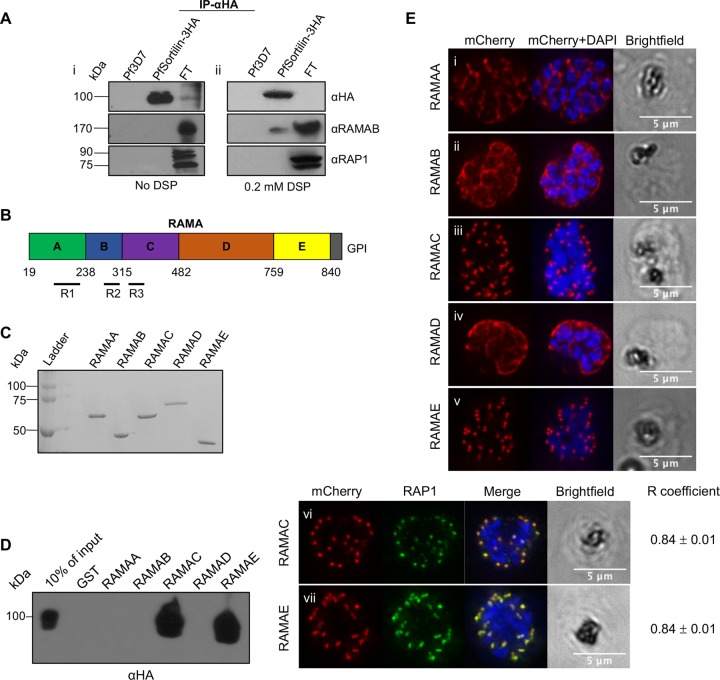
Regions of RAMA interacting with PfSortilin are sufficient for trafficking to the rhoptries. (A) Immunoprecipitation (IP) with anti-HA (αHA) shows that PfSortilin-3HA pulls down RAMA but only from DSP-cross-linked parasite lysates. FT, flow through. (B) Schematic of RAMA showing each of the five regions (RAMAA to RAMAE) that were used for further study. (C) Production of recombinant RAMA regions. Coomassie blue-stained gel showing the purification of the recombinant GST-RAMA fragments. (D) *In vitro* pulldown shows that recombinantly expressed RAMAC and RAMAE interacts with PfSortilin-3HA. The results shown are representative of two experiments using two different protein preparations and two different parasite extracts. (E) mCherry-RAMAC and mCherry-RAMAE fusion proteins traffic to the rhoptries, as confirmed by colocalization with RAP1, while RAMAA, RAMAB, and RAMAD fusions are trafficked to the parasitophorous vacuole (PV), the default destination for proteins harboring a signal sequence. The *R* coefficient values are means ± standard errors. The numbers of cells analyzed (*n*) are as follows: *n* = 10 for both RAMAC versus RAP1 and RAMAE versus RAP1.

Our previous results suggested that the soluble rhoptry bulb protein RAP1 was escorted from the Golgi apparatus to the rhoptries by the GPI-anchored protein RAMA. This led us to speculate on the existence of a putative transmembrane escort protein interacting with both the intraluminal RAP1-RAMA complex and the cytoplasmic trafficking machinery (11). To determine whether PfSortilin was interacting with RAMA and RAP1, we performed an anti-HA immunoprecipitation (IP) on protein extracts from the PfSortilin-3HA-tagged parasite line, but subsequent Western blots probed with anti-RAP1 and anti-RAMA antibodies showed that neither protein could be pulled down (Fig. 3Ai). To try to capture potential transient interactions between PfSortilin-3HA, RAMA, and RAP1, we cross-linked the parasites using the amine-reactive and membrane-permeable cross-linker dithiobis succinimidyl propionate (DSP) before solubilization and IP (24, 25). As seen in Fig. 3Aii, a small amount of RAMA can be pulled down with PfSortilin-3HA under these conditions. This demonstrates that a fraction of the total amount of RAMA interacts with PfSortilin-3HA *in vivo*. The absence of pulled-down RAP1 mirrors previous observations with RAMA-RAP1 immunoprecipitations: interaction was observed only when recombinant protein and not parasite lysate was used, suggesting that these interactions are transient (11, 26–28). To better define which regions of RAMA were binding to PfSortilin, we examined whether recombinantly expressed RAMA could pull down PfSortilin-3HA. As RAMA is a large protein of around 170 kDa (Fig. 3B), we expressed and purified five different regions encompassing the whole protein (RAMAA to RAMAE), excluding the signal peptide and the GPI anchor sequence (Fig. 3C). These regions were incubated with parasite lysates from the PfSortilin-3HA-tagged line, and as seen in Fig. 3D, RAMAC and RAMAE were both able to pull down PfSortilin-3HA specifically. Interestingly, RAMAC contains 11 imperfect repeats of the sequence EE(S/F)KN. Repeats are found in numerous *Plasmodium* proteins and are sometimes involved in mediating protein-protein interactions (29) so perhaps the RAMAC repeats are important for binding to PfSortilin. Of interest, RAMAE is also the region that we previously demonstrated interacts with RAP1 (11) and has also been shown to bind to an unidentified receptor on the surface of red blood cells (30). BLAST analysis of both RAMAC and RAMAE regions on the *P. falciparum* genome did not recover homologous regions in proteins other than RAMA (results not shown).

Given these results, we investigated whether the regions of RAMA that interact with PfSortilin are also important for trafficking to the rhoptries. Accordingly, wild-type (WT) *P. falciparum* 3D7 parasites were transfected with constructs expressing each region of RAMA as a fusion protein with an N-terminal mCherry reporter containing a signal peptide to allow entry into the secretory pathway. Examination of the transfectants showed that RAMAA, RAMAB, and RAMAD were found in the parasitophorous vacuole (PV), the default destination for proteins harboring a signal sequence (Fig. 3Ei, ii, and iv). In the case of RAMAC and RAMAE, a punctate pattern suggestive of an apical location was obtained (Fig. 3Eiii and v). Almost complete overlap between either RAMAC and RAMAE labeling with RAP1 by IFA confirmed their rhoptry localization (Fig. 3Evi and vii, *R* coefficient of 0.84 ± 0.01 for both RAMAC versus RAP1 and RAMAE versus RAP1). To summarize, these results show that PfSortilin interacts with RAMA *in vivo* and with specific portions of RAMA *in vitro* that are sufficient to localize a fluorescent reporter to the rhoptries. This therefore suggests that PfSortilin could potentially be the escorter that transports the RAMA-RAP1 complex from the Golgi apparatus to the rhoptries.

### Conclusions.

Our initial hypothesis was that the differential trafficking of apical complex proteins was mediated by a clustering mechanism at the Golgi apparatus whereby escorter proteins specific for the different apical organelles would bind their respective cargo for packaging into transport vesicles (11). Our findings that PfSortilin interacts with regions of RAMA sufficient for localization to rhoptries provide support for sortilin’s potential role as a protein escorter to this organelle. In other eukaryotic cells, binding of the cytoplasmic tail of sortilin to the intracellular trafficking machinery provides specificity and ensures the targeting of the cargo-containing vesicles to the proper organelles (14). It is tempting to speculate that PfSortilin could interact with Rab11A and AP-1, as these proteins have been hypothesized to be involved in vesicular fusion at the rhoptry membrane (9, 10). Intriguingly, TgSortilin has been shown to not only be critical for the trafficking of rhoptry proteins but also of micronemal protein, and its knockdown led to fully formed parasites lacking these organelles. Whether the same is true in *P. falciparum* remains to be seen and will require the use of parasite strains where PfSortilin expression can be conditionally regulated.

## MATERIALS AND METHODS

### Ethics statement.

This study was approved by the Canadian Blood Services (CBS) research ethics board (project 2015.001) and by the CHU de Québec institutional review board (IRB) (project 2015-2230, B14-12-2230, SIRUL 104595). Written consent was obtained by the CBS for all study participants. Participants were informed about the study before providing consent. All experiments were performed in accordance with relevant guidelines and regulations.

### Parasite culture.

*P. falciparum* 3D7 parasites were maintained in human O+ erythrocytes at a hematocrit of 4% with 0.5% (wt/vol) Albumax (Invitrogen) in RPMI 1640 medium (Life Tech). *P. falciparum* 3D7 parasites were originally obtained from David Walliker at Edinburgh University. Cultures were synchronized by incubation with 0.3 M alanine for 10 min (31).

### Cloning and transfection.

All primers used are listed in Table 1, and all plasmids were sequenced and analyzed before transfection. For the PfSortilin-3HA-tagged line, a fragment of 1 kb upstream of the stop codon of the *PfSortilin* gene was amplified from *P. falciparum* 3D7 cDNA and cloned into the BglII-PstI site of the pHA3 vector (32). Wild-type (WT) *P. falciparum* 3D7 parasites were transfected with the PfSortilin-3HA plasmid, and integrants were selected and cloned as described previously (33). Briefly, *P. falciparum* 3D7 parasites were transfected with 100 μg of purified plasmid DNA (Promega). Integrated parasites were selected using 20 nM WR99210 (Jacobus Pharma).

**TABLE 1:** List of primers used in this study

Primer^a^	Sequence^b^	Restriction site
Sortilin 1306 fw	5′ ATAAGATCTGAGACAAATACAGAAAAAAG 3′	BglII
Sortilin stopless rev	5′ ATACTGCAGCTAATAATTCAATATTATCAGC 3′	PstI
Rab7 fw	5′ GCCCTAGGATGTCAAATAAAAAAAGAACCATATTAAAAG 3′	AvrII
Rab7 rev	5′ TACCTCGAGTTAACAACAACGACTTTTG 3′	XhoI
RamaA fw	5′ ATAGGATCCGAACAAATAAAAAATGGTATAAGC 3′	BamHI
RamaA rev	5′ ATACTCGAGGCTATCATCGTATTCGTCAG 3′	XhoI
RamaB fw	5′ ATAGGATCCAGCGAAGAATATGATTACGAC 3′	BamHI
RamaB rev	5′ ATACTCGAGATATTTCATTTGTTCGTCTTTCATCTC 3′	XhoI
RamaC fw	5′ ATAGGATCCGTGATGAAAGATGAAGAGATG 3′	BamHI
RamaC rev	5′ ATACTCGAGTTTCTCATCATTTTGTAAGAAACT 3′	XhoI
RamaD fw	5′ ATAGGATCCAAAAAAATGGTCTTTTATGATTTATACAAGC 3′	BamHI
RamaD rev	5′ ATACTCGAGATCGAAAATTTTATTATTATTTTC 3′	XhoI
RamaE fw	5′ ATAGGATCCGATAATAAATTTGTAGCACATAAA 3′	BamHI
RamaE rev	5′ ATACTCGAGGCTTGACTTATTTCCATTTTC 3′	XhoI
RamaA fw	5′ ATAACGCGTGAACAAATAAAAAATGGTATAAGC 3′	MluI
RamaA rev	5′ ATAACTAGTGCTATCATCGTATTCGTCAG 3′	SpeI
RamaB fw	5′ ATAACGCGTAGCGAAGAATATGATTACGAC 3′	MluI
RamaB rev	5′ ATAACTAGTATATTTCATTTGTTCGTCTTTCATCTC 3′	SpeI
RamaC fw	5′ ATAACGCGTGTGATGAAAGATGAAGAGATG 3′	MluI
RamaC rev	5′ ATAACTAGTTTTCTCATCATTTTGTAAGAAACT 3′	SpeI
RamaD fw	5′ ATAACGCGTAAAAAAATGGTCTTTTATGATTTATACAAGC 3′	MluI
RamaD rev	5′ ATAACTAGTATCGAAAATTTTATTATTATTTTC 3′	SpeI
RamaE fw	5′ ATAACGCGTGATAATAAATTTGTAGCACATAAA 3′	MluI
RamaE rev	5′ ATAACTAGTGCTTGACTTATTTCCATTTTC 3′	SpeI

a fw, forward; rev, reverse.

b Restriction sites are underlined.

The RAMA fragments were PCR amplified from *P. falciparum* cDNA and cloned into the MluI-SpeI sites in the pTET-MSP2p-SP-mCherry which allows schizont-stage expression and entry into the secretory pathway. The transfectants were kept on 0.5 µg/ml anhydrotetracycline to prevent transgene expression.

To generate the green fluorescent protein (GFP)-Rab7 constructs, Rab6 was removed from pARL-GFP-Rab6 (34) and replaced by full-length Rab7 amplified from cDNA and digested with AvrII-XhoI.

### Southern blot.

Integration of the PfSortilin-3HA plasmid at the proper locus was confirmed by Southern blotting by standard procedures. Briefly, genomic DNA (gDNA) was extracted from parasites using the blood genomic DNA extraction kit (Sigma). For each parasite line, 10 µg of gDNA was digested with HindIII-EcoRV (PfSortilin-3HA). Digested DNA fragments were separated on 0.7% (wt/vol) agarose gel and then transferred to a Hybond N+ membrane (GE) and hybridized.

### Western blotting.

Saponin-extracted parasites from a highly synchronous PfSortilin-3HA-tagged line were harvested at the ring, early trophozoite, late trophozoite, and schizont stages. Proteins were then separated on 7% (wt/vol) SDS-polyacrylamide gel under reducing conditions and transferred to a polyvinylidene difluoride (PVDF) membrane (Millipore). The membrane was blocked in 4% (wt/vol) milk in Tris-buffered saline with Tween 20 (TBS-T). The antibodies used were mouse monoclonal anti-HA (clone HA.C5; Cedarlane) (diluted 1:200), mouse monoclonal anti-aldolase (MB720; Immunology Consultants Inc.) (1:1,000), rabbit polyclonal anti-PfHSP70 (SPC-186C; StressMarq Bioscience Inc.) (1:20,000), mouse monoclonal anti-PfRON4 (1:2,000) (35), rabbit polyclonal anti-RAMAB (1:1,000) (30), and mouse monoclonal anti-RAP1 (1:3,000) (36). Appropriate horseradish peroxidase (HRP)-coupled secondary antibodies were used, and immunoblots were developed using ECL (Bio-Rad).

### Microscopy.

Fluorescence microscopy was performed as previously described (37) using a GE Applied Precision Deltavision Elite microscope with a 100× 1.4-numerical-aperture (NA) objective and with a scientific complementary metal oxide semiconductor (sCMOS) camera and deconvolved with the SoftWorx software. For immunofluorescence assays (IFA), parasites were fixed with 4% paraformaldehyde (ProSciTech). After the slides were blocked with 3% bovine serum albumin (BSA) (fraction V; EMD), they were probed with combinations of antibodies: mouse anti-HA (clone HA.C5; Cedarlane; diluted 1:1,000), rabbit anti-HA (Abm; 1:1,000), rabbit anti-ERD2 (MRA-72; 1:1,000) (20), rabbit anti-AMA1 (1:2,000) (38), mouse anti-RAP1 (1:3,000), rabbit anti-RAMAB (1:1,000) (30), rabbit anti-BiP (1:500) (S. Absalon and J. Dvorin, unpublished data), and mouse monoclonal anti-AMA1 (clone 1F9; 1:500) (39). Primary antibodies were probed with Alexa Fluor 594-labeled anti-rabbit IgG or anti-mouse IgG (Molecular Probes) and Alexa Fluor 488-labeled anti-rabbit IgG or anti-mouse IgG (Cell Signaling). Slides were mounted with 4′,6-diamidino-2-phenylindole dihydrochloride (DAPI) (Invitrogen) (100 ng/µl) in VectaShield (Vector Labs) or ProLong Gold antifade mountant (Molecular Probes). Pearson’s correlation coefficient between Alexa Fluor 488 and Alexa Fluor 594 channels were calculated on deconvolved regions of interests of image stacks, including zero-zero pixels and without thresholding using the SoftWorx software (GE). Data were analyzed for statistical significance using one-way analysis of variance (ANOVA) followed by a Tukey multiple-comparison test. Chromatic calibration of the microscope was performed prior to imaging experiments.

To image the parasites transfected with the pTET-MSP2p-SP-mCherry-RAMA constructs, anhydrotetracycline was removed from cultures 72 h prior to imaging to allow expression of the mCherry fusions.

### Recombinant protein expression and pulldown assay.

Fragments of RAMA (RAMAA to RAMAE) were expressed as recombinant protein fused to a glutathione *S*-transferase (GST) tag in *Escherichia coli*. RAMA-GST fusion proteins were purified using glutathione-agarose beads (Sigma). Protein expression was confirmed by Western blotting with a polyclonal rabbit anti-GST (catalog no. A190-122A; Bethyl Labs). Protein concentration was determined by using a Bradford assay kit (Bio-Rad).

PfSortilin-3HA-tagged parasite pellets from saponin extracts were resuspended in lysis buffer (1% Triton X-100 [TX-100], 50 mM Tris [pH 7.4], 150 mM NaCl, 5 mM EDTA) in the presence of cOmplete protease inhibitor (Roche) and then lysed by mild sonication on ice. Proteins were extracted on ice for 45 min, and the insoluble material was separated by centrifugation.

For the pulldown assay, 100 µg of purified RAMA-GST bound on glutathione-agarose beads was incubated with the parasite lysate overnight at 4°C with rotation. Beads were then washed with wash buffer (20 mM Tris-HCl [pH 7.4], 150 mM NaCl) and resuspended in SDS sample buffer. Proteins bound to RAMA-GST beads were separated on a 7% (wt/vol) SDS-polyacrylamide gel under reducing conditions and blotted onto a PVDF membrane. PfSortilin-3HA was detected using a mouse monoclonal anti-HA antibody diluted 1:200 (clone HA.C5; Cedarlane).

To directly investigate protein interactions, immunoprecipitations followed by Western blotting were performed. Synchronized schizonts of the PfSortilin-3HA-tagged and WT *P*. *falciparum* 3D7 lines were saponin extracted and lysed in 1% Triton X-100 buffer with cOmplete protein inhibitor (Roche). Immunoprecipitation was performed with anti-HA affinity matrix (Roche). Washed beads were resuspended directly in sample buffer, and interacting partners were analyzed by Western blotting using anti-RAP1 and RAMA antibodies. The flow through was kept and loaded as a control. For chemical cross-linking prior to parasite lysis and protein extraction, synchronized schizonts of the PfSortilin-3HA-tagged and WT *P*. *falciparum* 3D7 lines were incubated with 0.2 mM DSP (Thermo Fisher Scientific).
